# Factors related to cell phone use while driving based on the Theory of Planned Behavior among university students in Kerman, Iran

**DOI:** 10.5249/jivr.v11i2.1120

**Published:** 2019-07

**Authors:** Narges Khanjani, Leila Tavakkoli, Shahrzad Bazargan-Hejazi

**Affiliations:** ^*a*^ Neurology Research Center, Kerman University of Medical Sciences, Kerman, Iran.; ^*b*^ Department of Biostatistics and Epidemiology, School of Public Health, Kerman University of Medical Sciences, Kerman, Iran.; ^*c*^ Department of Psychiatry, Charles R. Drew University of Medicine and Science& David Geffen School of Medicine at University of California at Los Angeles (UCLA), Los Angeles, USA.

**Keywords:** Texting and reading While driving, Cell phone use, Distracted driving, Theory of Planned Behavior

## Abstract

**Background:**

There is a paucity of research examining the pattern of and factors that influence cell phone use while driving in Iran. This study aimed to report the prevalence of and factors related to cell phone use while driving based on the Theory of Planned Behavior, among a sample of college students in Iran.

**Methods:**

This cross-sectional study was conducted in Kerman, Iran, in 2016. Participants volunteered to participate after the study was advertised. We used the Texting While Driving questionnaire (TWD) based on the theory of planned behavior for data collection and used SPSS 22 to calculate linear regression and Pearson correlation coefficients.

**Results:**

Two hundred and fifty-seven (n=257) students completed the questionnaire. Participants were between the ages of 18 and 47 years; 54.4% were females; 52.1% were single; 47.1% were undergraduate students, and 47.9% did not work. There was a significant relationship between intention toward sending and reading text messages while driving and gender, attitude, subjective norm, Perceived Behavioral Control (PBC), and moral norm. Also, there was a significant relation between willingness to send and read text messages while driving and PBC, subjective norm, group norm, and moral norm. For intention, personal factors were involved, and willingness was affected by both personal factors and friend/peer groups.

**Conclusions:**

Texting while driving is prevalent among college students in Kerman; and intention and willingness to do this, is affected by several factors. Our findings suggest needs for the individual and peer- group training, and increasing awareness about the dangers of using cell phones while driving among this sample.

## Introduction

According to the World Health Organization report (WHO), road traffic crashes kill about 1.25 million people a year, and these injuries are the leading cause of death in the young, 15 to 29 years old age group.^1,2^ Also, between 20 to more than 50 million people suffer from non-fatal road traffic injuries annually. Developing countries experience 90% of the global road crash fatalities. ^1,2^ In Iran as a developing country, mortality from road traffic injuries is the second cause of death after cardiovascular diseases.^3^

Driver distraction is a major risk factor for crashes.^4,5^ The Center for Disease Control (CDC) reported that in the USA, more than eight people die and more than 1100 get injured in road crashes, because of driver distraction each day. The three main types of distraction are taking eyes off the road, taking hands off the wheel and taking the mind off driving.^6^

Although, many factors can cause driver distraction, using cell phones while driving and especially texting while driving can cause a significant reduction in performance of young drivers, and this has increased public concern for road safety.^2^ Distraction is different from inattention, and any internal and external factor can cause distraction,^7^ which eventually lead to human error.^8^ Previous studies showed some behavioral factors could affect both sending and reading text messages while driving.^9,10^

Mobile phone use increases distraction. According to the WHO report, the risk of crashes for drivers who use cell phones is four times more than others who do not use it.^1^ Likewise, studies have shown that the risk of getting involved in road crashes is much more for drivers who use cell phones than others.^11^ Also using a cell phone with or without a hands-free device increases the driver's reaction time therefore the risk of crash^12^ not only for the driver but also for the pedestrians.^13-15^ It has also been reported that young adults who used cell phones while driving, were more likely to have other risky behaviors during driving as well.^2,15^

According to the United Nations International Children's Emergency Fund^16^ report, about 90% of road traffic deaths occur in developing countries, and the rate of road crashes in Iran is 20 times more than the average in the world.^16^ Although, mobile phone use while driving increases the risk of a crash, four to nine times, ^17,2^ the extent of the risk is related to the type of usage.^18^ In Iran, studies have shown that the most frequent cause of road traffic crashes was not paying attention to the road in front^19,20^ a distraction that often is caused by using mobile phones.^21^ According to Ashrafi Asgarabad et al., the use of mobile phones in Kerman, Iran is generally high, and much higher in younger age groups, compared to the developed countries.^22^

Meanwhile, Khosravi Shadmani et al., report that cell phone use is one of the most avoidable risk factors for serious road traffic crashes in Iran.^23^ A few studies in Iran have focused, specifically on the prevalence of cell phone use while driving and its related risk factors. This paucity in research exists despite the high rate of traffic-related mortality in Iran.^20^ Also, traffic-related crashes are a health dilemma and the first cause of mortality in Iranian youths.^20,24^ Students are a group that is involved in both driving and text messaging. Studying their behavior can help identify some driving safety parameters. The present study aims to investigate patterns of, and factors associated with using cell phones while driving among college students in one of the largest province of Iran, Kerman. 

## Methods 

**Recruitment and Study Sample**

The current investigation was a cross-sectional study conducted among college students in Kerman, Iran, in 2016. We recruited students through flyers and advertisements. They were eligible to participate in the study if they had enrolled in an academic program of one of the universities in Kerman including Kerman University of Medical Sciences, Bahonar University, Payam Noor University, The Applied Sciences University. In addition, eligible students had to have a current driver's license, own a cell phone, driven a vehicle at least once a week and be willing to fill out the study questionnaire as a way of consenting to the study. Students were excluded from the study if they did not meet these eligibility criteria. Two hundred fifty-seven (n= 257) students met the study eligibility and enrolled in the study.

**Study Instrument**

We used the Texting While Driving (TWD), and demographic questionnaire for this study. The TWD questionnaire was developed based on the theory of planned behavior (TPB) and was validated and used among U.S. college students.^25,9,26^ The self-administered questionnaire consisted of 50-theory-based items in addition to several socio-demographic and driving related items including gender, marital status, participant's academic degree, occupation, location and province of residence, type of driver's license, type of vehicle gear, and the number of children. 

The TPB constructs in the questionnaire included intention, which refers to the inclination to engage in the behavior; attitude, which is belief about the outcome of the behavior and evaluation of the outcomes; subjective norms, which refers to the motivation to comply with the expectations of one's significant others; perceived behavioral control, which is beliefs about factors that can either facilitate or hinder the performance of the behavior, and the perceived power of these factors. Group norm refers to the context-specific attitudes and behaviors of group members concerning approving or disapproving of certain behaviors. It highlights the influence of one's referent group on one's decision-making, as opposed to the subjective norm, which implies social pressure from all people who are important to oneself (significant others).^9,26^

We assessed the intention, attitude, and subjective norms towards sending and reading TWD by using 3-item, 7-point Likert scale responses. Perceived Behavioral Control (PBC) was assessed by using 2-item, 7-point Likert scale responses. We also measured group norm as a 4-item, 7-point Likert scale, and moral norm as a 3-item, 7-point Likert scale. The Cronbach's alpha for each scale was calculated separately and ranged from 0.72 to 0.90.^25^

Hosseini et al. have reported the validity and reliability of this questionnaire in Farsi.^27^ The Cronbach's alpha (internal consistency) and the intra-class correlation for all scales in "send" and "read" text while driving ranged from 0.71 to 0.79 and 0.79 to 0.83, respectively.^27^ The construct of the moral norm as an extension of TPB has been widely used in studying driving behaviors. In general, it is hypothesized that feeling moral obligation and regret can prevent an individual's intention to engage in risky driving behavior.^25,9,24^ The behavioral willingness construct from the Prototype/Willingness Model (PWM), may predict behavior, in addition to other TPB constructs.^28^ The "willingness variable" may account for the irrationality of health-risk behaviors in young people. It may also apply to behaviors that are more reactive (non-intentional) in nature, such as getting pregnant, acquiring STDs, or drunk driving.^29^ In order to analyze analyze the data, we used SPSS version 22 and calculated linear regression and Pearson correlations. Statistical significance was established at p ≤ 0.05.

## Results

**Descriptive Statistics**

Two hundred fifty-seven (n= 257) students in an age range from 18 to 47 completed the questionnaire. The mean age of participants was 26.79±6.35 years. The majority were female (54.4%), and single (52.1%). Slightly over 47 % of the participants were undergraduate, and 47.9% did not work. Most students drove cars with manual gear (80.2%) and held a light vehicle (type 2) driver's license (46.7%). Most students lived in Kerman Province (75.1%) and the urban area (96.1%) (Table 1).

**Table 1:** Demographic characteristics of the participants.

Variables	Frequency (%)	
	
Gender	Male	114 (44.4)
	Female	140 (54.5)
	Total	254 (98.8)
	Unknown	3 (1.2)
Marital status	Married	110 (42.8)
	Single	134 (52.1)
	Separated	4 (1.6)
	Divorced	5 (1.9)
	Widow/Widower	2 (0.8)
	Total	256 (99.6)
	Unknown	2 (0.8)
Students degree	Graduate Diploma Student	3 (1.2)
	Bachelor Student	121 (47.1)
	Masters Student	70 (27.2)
	MD Student	30 (11.7)
	PhD Student	11 (4.3)
	Medical Resident	19 (7.4)
	Others	2(0.8)
	Total	256 (99.6)
	Unknown	1 (0.4)
Occupation	Full time job	45 (17.5)
	Part time job	51 (19.8)
	Self-employment	5 (1.9)
	Without joband looking for it	21 (8.2)
	Without job and not looking for it	125 (47.9)
	Others	9 (2.6)
	Total	253 (98.4)
	Unknown	4 (1.6)
Location of residence	Rural area	8 (3.1)
	Urban area	247 (96.1)
	Total	255 (99.2)
	Unknown	2 (0.8)
Province of residence	Kerman	193 (75.1)
	Others	55 (21.4)
	Total	248 (96.5)
	Unknown	9 (3.5)
Type of driver's license	Recent type 3license	69 (26.8)
	Recent type 2 license	120 (46.7)
	Old type 2 license	42 (16.3)
	Type 1	5 (1.9)
	Others	4 (1.6)
	Total	240 (93.4)
	Unknown	17 (6.6)
Type of vehicle gear	Manual	206 (80.2)
	Automatic	41 (16)
	Total	247 (96.1)
	Unknown	10 (3.9)
Has Children?	Yes	90 (35.0)
	No	165 (64.2)
	Total	255 (99.2)
	Unknown	2 (0.8)
Involved in any multivehicle accidents or crashes as a driver	No	182 (70.8)
	Yes, resulted in no injury	53 (20.6)
	Yes, resulted in minor injury	9 (3.5)
	Yes, resulted in major injury	8 (3.1)
	Yes, resulted in death	4 (1.6)
	Total	256 (99.6)
	Unknown	1 (0.4)
Stopped by the police for using cell phone while driving	Yes	59 (23)
	No	190 (73.9)
	Not applicable	4 (1.6)
	Total	253 (98.4)
	Unknown	4 (1.6)
Sent or Read text messages while driving within the past 30 days	Never	59 (23.0)
	A few times	89 (34.6)
	Several times a day	66 (25.7)
	Many times a day	21 (8.2)
	Every day	19 (7.4)
	Total	254 (98.8)
	Unknown	3 (1.2)
Talked on cellphone while driving within the past 30 days	Never	91 (35.4)
	A few times	81 (31.5)
	Several times a day	52 (20.2)
	Many times a day	14 (5.4)
	Every day	16 (6.2)
	Total	254 (98.8)
	Unknown	3 (1.2)

The median, mean, and standard error of messages sent by participants per day were 12, 225.7, and 943.2, respectively. The median, mean, and standard error of messages read by students per day were 15, 67.5, and 2991.8, respectively. The median, mean, and standard error of messages received by participants per day were 20, 693.9, and 3030.8, respectively. 

On average, the participants had held a driver's license for 5.84±4.61 years, owned a cell phone for 9.06±3.78 years, and drove 17.92±28.48 hours per week. At least, 63.4% of the participants had talked on their cell phone while driving within the past 30 days and 75.8% had read or sent text messages while driving within the last month (Table 1). Additionally, 59 (23%) reported that they had been stopped by the police at least once for using their cell phone while driving, and 74 (31.8%) had been involved in a crash as the driver. A few crashes had caused death (Table 1).

**Analytical Statistics**

There was a strong direct significant correlation (p<0.001) between sending and reading text messages while driving for attitude (r=0.895), subjective norm (r=0.909), perceived behavioral control (r=0.923), intention (r=0.734), group norm (r=0.870), moral norm (r=0.958) and willingness (r=0.542) (Table 2).

**Table 2:** Correlations, mean and standard deviation in sending and reading text messages while driving.

Variable(Scored from 1 to 7)	n	r	P-Value	Send		Read	
				Mean	SD	Mean	SD
**Attitude**	242	0.895	<0.001	2.44	1.67	2.55	1.78
**Subjective norm**	251	0.909	<0.001	2.67	1.38	2.67	1.42
PBC*	251	0.923	<0.001	3.86	1.63	3.89	1.62
**Intention**	252	0.734	<0.001	2.65	1.44	2.78	1.42
**Group norm**	250	0.870	<0.001	2.11	0.87	2.11	0.87
**Moral norm**	250	0.958	<0.001	5.09	1.72	5.06	1.71
**Willingness**	251	0.542	<0.001	4.35	1.40	4.51	1.42

*PBC=perceived behavioral control

The mean score of attitudes was the lowest, which shows the negative attitude of participants regarding sending (2.44) and reading (2.55) text messages while driving. The mean scores of sending and reading while driving for subjective norm, intention, and group norm were low, as well. These findings point to the low attitude, intention, subjective norm, and group norm of the participants towards sending and reading text messages while driving (Table 2).

The participants' high mean score for the moral norm towards sending (5.09) and reading (5.06) text messages while driving suggests that they considered using cell phones while driving is morally wrong. The mean willingness score of 4.35 for sending and 4.51 for reading shows high willingness toward sending and reading text while driving in this sample. (Table 2).

There was a significant direct correlation (p<0.001) between the mean of the moral norm and PBC, although it was weak; r=0.224 and r=0.261, for sending and reading text messages, respectively. The higher the score of the moral norm, the higher the score of PBC (Table 3 and 4).

There was also a significant direct correlation (p<0.001) between subjective norm and group norm, r=0.435 and r=0.424 for sending and reading text messages, respectively. We also found a significant direct correlation between willingness and intention (r=0.260) for both reading and sending, although weak. These findings meant students who were more willing to use a mobile phone while driving also had a higher intention to use it (Table 3 and 4).

**Table 3:** Bivariate correlations coefficients for sending text messages while driving.

Variable	Attitude	Subjective norm	PBC	Intention	Group norm	Moral norm	Willingness
**Attitude**	1.000	0.189**	0.108	0.266***	0.155**	-0.204**	-0.002
**Subjective norm**		1.000	0.344***	0.676***	0.435***	-0.146*	0.341***
**PBC**			1.000	0.420***	0.179**	0.224***	0.310***
**Intention**				1.000	0.313***	-0.105	0.260***
**Group norm**					1.000	-0.105	0.359***
**Moral norm**						1.000	0.157*
**Willingness**							1.000

*p-value <0.05 **p-value <0.01 ***p-value <0.001

**Table 4:** Bivariate correlations coefficients for reading text messages while driving.

Variable	Attitude	Subjective norm	PBC	Intention	Group norm	Moral norm	Willingness
Attitude	1.000	0.228***	0.053	0.370***	0.193**	-0.252***	0.023
Subjective norm		1.000	0.354***	0.803***	0.424***	-0.148*	0.279***
PBC			1.000	0.385***	0.215**	0.261***	0.287***
Intention				1.000	0.403***	-0.193**	0.260***
Group norm					1.000	-0.058	0.331***
Moral norm						1.000	0.206**
1.000							Willingness

*p-value <0.05 **p-value <0.01 ***p-value <0.001

Results of multiple regression analysis showed a significant relationship between intention toward sending text messages and occupation (β=-0.154, p=0.001), attitude (β=0.127, p=0.005), subjective norm (β=0.573, p<0.001) and PBC (β=0.224, p<0.001). The final model, including these four variables, explained 54% of intention toward sending text messages while driving (Table 5 ).

**Table 5:** Multiple regression analysis: Intention to send and read text messages while driving.

	Variable	Beta Enter	CI for Beta Enter	P-value for Beta Enter	Adj Rsquare	Beta BLRT*	CI for Beta BLRT	P-value for Beta BLRT	Adj Rsquare
Sending texts	Gender	-0.093	-1.678, 0.045	0.063		-0.067	-1.406, 0.222	0.153	
	Age	0.005	-0.075, 0.081	-0.937		---	---	---	
	Marital status	-0.025	-1.116, 0.679	0.632	0.533	---	---	---	0.540
	Student degree	-0.020	-1.047, 0.700	0.696		---	---	---	
	Occupation	-0.134	-2.131, -0.268	0.012		-0.154	-2.158, -0.606	0.001	
	Attitude	0.102	0.005, 0.169	0.037		0.127	0.034, 0.188	0.005	
	Subjective norm	0.552	0.469, 0.697	<0.001		0.573	0.506, 0.704	<0.001	
	PBC	0.218	0.154, 0.424	<0.001		0.224	0.176, 0.424	<0.001	
	Group norm	0.020	-0.100, 0.150	0.694		---	---	---	
	Moral norm	-0.057	-0.133, 0.036	0.258		---	---	---	
Reading texts	Gender	-0.084	-1.456, -0.017	0.056		-0.106	-1.578, -0.240	0.008	
	Age	0.048	-0.034, 0.100	0.329		0.051	0.084, 0.893	0.208	
	Marital status	0.024	-0.541, 0.965	0.579	0.667	---	---	---	0.680
	Student degree	-0.018	-0.896, 0.592	0.687		---	---	---	
	Occupation	-0.047	-1.200, 0.372	0.300		---	---	---	
	Attitude	0.130	0.038, 0.171	0.002		0.132	0.043, 0.169	0.001	
	Subjective norm	0.671	0.582, 0.766	<0.001		0.685	0.602, 0.768	<0.001	
	PBC	0.148	0.078, 0.309	0.001		0.143	0.075, 0.301	0.001	
	Group norm	0.034	-0.064, 0.147	0.441		---	---	---	
	Moral norm	-0.100	-0.160, -0.012	0.023		-0.092	-0.148, -0.008	0.029	

* Backward Likelihood Ratio Test

Intention toward reading text messages was lower in women (p=0.008) and there was also a significant relation between intention and attitude (β=0.132, p=0.001), subjective norm (β=0.685, p<0.001), PBC (β=0.143, p=0.001) and moral norm (β=-0.092, p=0.029). The final model with these five variables explained 68% of intention towards reading text messages while driving (Table 5).

Furthermore, we found a significant relation between willingness to send text messages and PBC (β=0.130, p=0.043), subjective norm (β=0.220, p=0.001), group norm (β=0.271, p<0.001) and moral norm (β=0.154, p=0.011). The final model with these four variables explained 24% of willingness towards sending text messages while driving (Table 6). The results also showed a significant relation between willingness toward reading text messages while driving and PBC (β=0.141, p=0.023), group norm (β=0.348, p<0.001) and moral norm (β=0.249, p<0.001), and the final model with these two variables explained 23% of the variance (Table 6).

**Table 6:** Multiple regression analysis: Willingness to send and read text messages while driving.

	Variable	Beta Enter	CI for Beta Enter	P-value for Beta Enter	Adj Rsquare	Beta BLRT*	CI for Beta BLRT	P-value for Beta BLRT	Adj Rsquare
Sending texts	Gender	-0.053	-1.009, 0.420	0.418		---	---	---	
	Age	0.056	-0.040, 0.089	0.451		---	---	---	
	Marital status	0.105	-0.145, 1.335	0.115	0.230	0.074	-0.213, 1.049	0.193	0.240
	Student degree	0.032	-0.547, 0.900	0.631		---	---	---	
	Occupation	0.045	-0.510, 1.030	0.507		---	---	---	
	Attitude	-0.091	-0.118, 0.018	0.148		-0.083	-0.111, 0.019	0.166	
	Subjective norm	0.195	0.038, 0.226	0.006		0.220	0.058, 0.233	0.001	
	PBC	0.146	0.012, 0.234	0.030		0.130	0.004, 0.218	0.043	
	Group norm	0.266	0.100, 0.316	<0.001		0.271	0.116, 0.317	<0.001	
	Moral norm	0.118	-0.005, 0.135	0.069		0.154	0.020, 0.151	0.011	
Reading texts	Gender	-0.121	-1.438, 0.060	0.071		-0.103	-1.24, -0.062	0.076	
	Age	0.143	-0.001, 0.135	0.54	0.228	0.099	-0.013, 0.104	0.126	
	Marital status	0.087	-0.264, 1.265	0.198		0.086	-0.237, 1.227	0.184	0.232
	Student degree	-0.030	-0.932, 0.588	0.656		---	---	---	
	Occupation	0.133	-0.015, 1.585	0.054		0.110	-0.038, 1.325	0.064	
	Attitude	-0.002	-0.068, 0.066	0.980		---	---	---	
	Subjective norm	0.095	-0.030, 0.157	0.181		0.099	-0.024, 0.158	0.149	
	PBC	0.114	-0.018, 0.217	0.098		0.141	0.017, 0.232	0.023	
	Group norm	0.282	0.123, 0.338	<0.001		0.348	0.192, 0.380	<0.001	
	Moral norm	0.198	0.038, 0.188	0.003		0.249	0.073, 0.208	<0.001	

## Discussion

Research has shown that using cell phones while driving (distractive driving) increases the probability of road crashes/near-crashes.^30,31^ There is, also, a significant relationbetween crash involvement and mobile phone use while driving.^17^

Our sample of college students in this study was relatively young, and the majority were female and single. Almost half of the sample were undergraduate students and did not work. Most students drove cars with manual gear, lived in urban areas of the Kerman province. 

More than half of the sample in the current study had talked on their cell phone while driving within the past 30 days, and about 1 in three students in the sample had read or sent text messages while driving within the last month. However, a small number of these students were stopped by the police, and nearly one in four experienced a car crash as a driver. Others reported the high prevalence of distracted driving. For example, a national survey in the U.S. in 2016 showed that 59% of drivers aged 19-24 years, sent a text message while driving at least once in a month.^32^ In a different study in the U.S. nearly 70% of the college students reported using a cell phone to send and read text message a few times during the last week, and only 27% reported being stopped by police for using cell phones while driving.^26^ Also, 10.7% of the U.S. sample had been involved in a multi-vehicle crash as the driver.^26^ The intention of sending text messages was lower in women than in men. Likewise, in a New Zealand study, male drivers more often reported using a cell phone while driving.^17^ However, female college school drivers in the U.S. study had significantly more intention for sending and reading text messages while driving.^26^

The high prevalence of TWD in the participants of the present study may be due to their young age and more risk-taking behaviors during this age.^33^ In one of the largest province of Iran (Kerman), the authors showed that fatal road crashes decreased as the drivers' age increased.^20^ In an earlier study, the authors reported that a higher percentage of younger drivers with fewer years of driving experience used cell phones while driving.^17^ Our findings also reveal that on average, this sample of college students was more apt to read texts than send while driving. Likewise, Tison et al. reported that their participants answered calls and read text messages while driving more often than making calls or sending text messages.^33^ A different study showed that while people believe talking on cell phones while driving is hazardous, they tend to do it, especially if they believe the received call is important.^10^ We also found that students believe that sending and reading text messages while driving is the wrong thing to do, yet they tend to do it. In another study, 91% of the U.S. college students reported texting while driving and even reported driving above speed limits (53%) and even drifting into other lanes while texting (55%). Although, they believed texting while driving is hazardous, and it should be prohibited.^34^

In the present study, occupation, attitude, subjective norm, and PBC explained 54% of intention toward sending a text while driving and these variables without occupation and with moral norm explained 68% of intention toward reading text while driving. Others investigators also have discussed the predictive roles of attitude, subjective norm, and PBC in intention towards sending and reading text messages while driving.^9,26^

The present study also showed PBC, subjective norm, group norm, and moral norm explained 24% of willingness toward sending messages. Moreover, PBC, group norm with moral norm explained 23% of willingness toward reading text messages while driving. These results show that friends and peers can influence willingness toward texting while driving. Also, there was a significant relationbetween intention and willingness toward texting while driving, similar to the earlier study. ^26^ It is important to acknowledge the limitations of our study. Since we conducted a one-time survey, it was difficult to establish any causal relationbetween TPB variables and the study outcome measures. However, this approach allowed us to collect our data relatively fast when compared with prospective studies. ^35^ Our convenient sampling design limits our ability to generalize the study findings to other college campuses. Use of self-reported data subjects our finding to recall bias and social desirability, especially remembering the frequency and period of texting while driving could have been challenging. Also, it is possible that students who were already engaged in high-risk driving, including distractive driving, did not volunteer to participate in the study. However, despite these limitations, our findings provide valuable information for future policy and educational interventions.

## Conclusion

Mobile phone use for reading and sending text messages while driving is prevalent among college students in Kerman. Intention and willingness for this, is affected by several factors. Personal factors are more involved in intention, while willingness ae affected by both personal factors and friend/peer groups. Our findings suggest the need to enhance awareness about the dangers of using cell phones while driving to improve safe driving among college students. More study is needed to delineate the benefits of peer training in transferring information and changing the attitude and perception of college students regarding the risks of distractive driving. Further multi-campus longitudinal studies are needed to better depict the causal associations and underlying mechanism of the associations among TPB constructs. 
